# Editorial: Integrating climate change into health education and promotion

**DOI:** 10.3389/fpubh.2026.2002212

**Published:** 2026-10-23

**Authors:** Anusha Govind, William Brett Perkison, Adelita Gonzales Cantu

**Affiliations:** 1 University of Texas Southwestern Medical Center, Dallas, TX, United States; 2 University of Texas Health Sciences Center in Houston School of Public Health, Houston, TX, United States; 3 The University of Texas Health Science Center San Antonio School of Nursing, San Antonio, TX, United States

**Keywords:** climate change, education innovation, health, health education, medicine

Climate change poses significant risks to global health, exacerbating chronic health conditions, influencing the spread of infectious diseases, and even affecting food and water availability and security. As these environmental changes and its associated health risks accelerate, there is an urgent need to incorporate climate change education into the health professional curriculum. A recent survey of United States medical students reported that 83.9% believed that climate change and its health effects should be added into medical school curriculum, while only 13% believed that their school offered this education (1). This Research Topic aims to equip our readers with diverse perspectives on curriculum structures and designs, including curriculum innovation, competency development, leadership training, and practice transformation.

The collection starts with an overarching narrative review by Maisoon, reviewing the numerous ways in which climate change and health education has been incorporated into medical training throughout the last two decades. The themes which she identified can be seen in the other manuscripts.

A major theme across this Research Topic covers innovative ways to rapidly introduce climate and health education using various curriculum structures. Baldwin et al. describe a small group medical student elective that uses case-based sessions along with expert educators to introduce a wide breath of introductory material on climate and health to interested medical students. Similarly, Newman et al. developed a didactic curriculum with incorporated case-based learning for pediatricians and medical students. They were able to broaden the audience by presenting it to all medical students during a mandatory Social Influences on Health session. Dalapati et al. created an interprofessional course for faculty and students using asynchronous virtual modules as well as in person sessions to teach climate mitigation and adaption efforts at all levels. Chamber's paper describes her design, implementation and evaluation of a curriculum specific for internal medicine residents across a wide breadth of climate and health topics. Benzekri et al. piloted Medicine for a Changing Planet, an online program using competency based clinical cases, with an important integration of planetary and One health The above manuscripts highlight the importance of using innovative platforms of pedagogy to target learners at different stages of training. These offer multiple pathways for implementation of such a climate curriculum into an existing program, depending on the program's bandwidth and structure.

Moving beyond just incorporation into the traditional health professions educational settings, multiple papers discuss the multidisciplinary aspect of climate as a component of health education. The paper by Kalwaney and Cerceo, describes nutrition education as an important, yet underutilized educational component in this arena. She discusses the importance of nutrition as a therapeutic intervention, the quality and quantity of which will be affected by climate change. Similarly, Jeschonnek et al. describe how occupational therapists can incorporate climate-related health understanding into sessions with their clients. Like the challenges noted of incorporating climate change education into a medical school or residency program identified in some papers above, they address challenges incorporating it into client facing OT sessions. A paper by Mueller et al. retrospectively reviewed how climate is linked to pharmaceuticals and prescribing practices, and the need for more emphasis on the integration of these topics into health professional curriculum. Even further than just healthcare disciplines, Moula et al. connect art-based interventions to climate education communications, through creative means including art festivals, theater performances, field trips and sustainability interventions. These manuscripts give us a broad outlook on the non-traditional ways in which climate and health education can be incorporated thoughtfully into other disciplines outside of standard medical didactics.

Another focus of manuscripts in this Research Topic includes assessment and evaluation. Moon et al. describe a multi-step assessment protocol for climate and health education in a pediatric residency program. They retrospectively reviewed climate related health communications between providers and patients and linked them with assessment activities using the ACGME competency framework.

Another prominent theme is leadership and advocacy. Bjorgaard and Barrera present a peer-led model for climate and health leadership development that emphasizes collective action and community engagement. Their work reminds us that climate-health education must extend beyond knowledge acquisition to include the development of leadership, communication, and advocacy skills necessary to address complex societal challenges.

Taken together, the articles in this Research Topic demonstrate that health professions education has moved beyond debating the need to address climate change and is now focused on how best to integrate climate-related knowledge and skills into education and practice. The contributions highlight practical strategies for designing curricula, implementing innovative teaching approaches, defining and assessing competencies, and fostering leadership in climate and health. Collectively, these examples provide a foundation for advancing climate and health education across institutions, educational settings, and health professions disciplines, while supporting the translation of learning into meaningful clinical, public health, and community practice.

As editors, we are encouraged by the diversity of approaches represented in this collection and by the growing recognition that climate change is fundamentally a health issue. We hope these contributions will serve as a resource for educators, clinicians, researchers, and policymakers working to prepare the next generation of health professionals to address the health impacts of a changing climate and to advance more sustainable, equitable health systems.
